# Investigating Knowledge and Attitude of Nursing Students Towards Iranian Traditional Medicine: Case Study: Universities of Tehran in 2012-2013

**DOI:** 10.5539/gjhs.v6n6p168

**Published:** 2014-07-29

**Authors:** Sahar Rabani Khorasgani, Leila Moghtadaie

**Affiliations:** 1 Faculty of Medicine, Tehran University of Medical Sciences, Tehran, Iran; 2 Faculty of Education and Psychology, University of Isfahan, Isfahan, Iran

**Keywords:** attitude, Iranian traditional medicine, knowledge, nursing

## Abstract

The present study aimed at Investigating the knowledge and attitude of Nursing Students towards Iranian Traditional Medicine in universities of Tehran in 2012-2013. 300 students of nursing studying at different universities in Tehran participated in this descriptive, cross-sectional study. The data was collected through a standard questionnaire with an acceptable validity and reliability. The questionnaire was made of five sections including demographic, general knowledge of the Iranian traditional medicine, general attitude towards it, resources of the Iranian traditional medicine and the barriers to it. The results revealed that general knowledge of the students about Iranian traditional medicine and complementary medicine is low. The attitude of the students towards including Iranian traditional medicine and complementary medicine in their curriculum is positive. General attitude of students towards Iranian traditional medicine is positive too. The majority of the participants had not passed any course on Iranian traditional medicine. There was no relationship between participants’ attitude towards Iranian traditional medicine and the number of semesters they had passed. Considering the participants’ positive attitude and their low level of knowledge, it seems necessary for the university policy makers to provide nursing students with different training courses on Iranian traditional medicine and complementary medicine in order to increase their knowledge.

## 1. Introduction

In 1978, World Health Organization (WHO) defined traditional medicine in different statements with the purpose of developing this medicine in this way: “Traditional medicine is a collection of all theoretical and applied sciences which are employed in diagnosis, prevention and treatment of physical, mental or social disorders and are transferred from one generation to another in written or spoken form” (WHO, 1978). From the past times, traditional medicine has been a part of people’s health system and has been accepted by them. It has become part of their culture and has played a significant role in cultural issues of their health. Traditional medicine can easily assist the usual medicine. Developing and propagating it can in fact be considered as respect to the culture and heritage of people all over the world (Haji Akhundi, Mirghazanfari, & Fathahi, 2010). Research in the last two decades reveals the spread of using traditional medicine methods even without consulting or coordinating with formal physicians (Schimff, 1997). This attitude towards traditional medicine has two dimensions: one is the attitude which has been created among the people and the other is the attitude which has been created in academic, university and governmental circles. This increase in interest and use of traditional and complementary medicine among people is the result of the change in social patterns, values and social wants which has been made in modern health care system in the last few decades. In order to encourage health care, we should exactly understand the attitudes and internal wants of the society (Nasseri, 2006). In line with the developments in medicine, nurses should improve their knowledge in this regard because lack of qualifications in doing such kinds of treatments or insufficient knowledge of their effects or even its dangers may bring about various problems for patients (Zeighami, 2006). In addition, developing correct training and educating qualified and interested medical force decrease the possibility of misuse of traditional medicine. Furthermore, in this way, the required force for undertaking research in this area is educated (Nasseri, 2005). Findings of recent research reveal that although the students believe in increasing tendency of people towards methods of complementary medicine, they are also worried because of lack of scientific evidence about possible effects or side effects of these methods; therefore, it has been suggested that these topics should be incorporated into students’ textbooks in order to provide them with enough information and prepare them to encounter with this issue (Chez, Jonas, & Crawford, 2001). General differences in students’ beliefs across different health-related fields of study urge the necessity of a multi-dimensional planning in order to improve knowledge of people in different medicine and health related disciplines (Baugniet, Boon, & Ostbye, 2000). Traditional medicine is still unknown among Iranian medical society although its knowledge is vital for regeneration and revival of medical society in Iran and the whole world and also for giving appropriate advice to the patients (Haji Akhundi et al., 2010). Despite the increasing use of the complementary medicine across the world, Iranian traditional medicine has not yet been considered despite its richness and high efficacy as an independent medical school among the schools of complementary medicine. In addition, although teaching Iranian traditional medicine has been started in Iranian universities, unfortunately, this training is not yet considered as part of their formal curriculums. Therefore, knowledge of students’ presuppositions about Iranian traditional medicine, and its effects and side effects is very important (Naghibi, Jalali, ZarAfshar, Ebadiani, & Karbakhsh, 2009). Nurses play an active role in advising treatment methods to patients and this advice is usually based on their own perceptions of the advantages of the method. Therefore, extensive studies for investigating academic needs of nurses in order to give the required advice to the patients and participate in the patient’s treatment process in cooperation with the physicians are essential (Xue, Zhang, Holroyd, & Suen, 2008). The purpose of the present study is to analyze nursing students’ knowledge and attitude towards Iranian traditional medicine and its application. The findings of this study may have implications for decision makers for incorporating the required educational programs related to Iranian traditional medicine in their curriculum.

### 1.1 Traditional Medicine

In order to make its motto of “*Health for all by 2000*” applicable, WHO attended to develop traditional medicine. In 1978, WHO in a statement defined traditional medicine in this way: “Traditional medicine is a collection of all theoretical and applied sciences which are employed in diagnosis, prevention and treatment of physical, mental or social disorders and are transferred from one generation to another in written or spoken form” (WHO, 1978). In 2002, WHO defined traditional medicine with more elaborations in the following way: “Traditional medicine is a general term which refers both to the systems of traditional medicine like traditional Chinese, Ayurveda, Indian medicine, Unani and Arabic medicine, and also to different methods of folk medicines. It includes different treatments with or without using drugs. In countries in which the system of medical services is based on the modern medicine, the term complementary medicine is usually used instead of traditional medicine (Nasseri, 2006).

### 1.2 Some Kinds of Traditional and Complementary Medicine

#### 1.2.1 Phytotherapy

It refers to the treatment of different physical or mental diseases by using herbs.

#### 1.2.2 Massage Therapy

It refers to massaging hands on soft tissues in order to enhance the health level. Among its effects, removing negative moods and pain, decreasing blood pressure and heart beat, and removing stress and depression can be mentioned (Moyer, Rounds, & Hunnum, 2004).

#### 1.2.3 Cupping

It refers to extracting blood from some parts of the body for curing the diseases. It consists of two types: wet and dry. In the dry type, through sucking, blood and liquid are led to the surface of the skin and then, extracted from the body. In the wet type, a wet, warm sponge is rubbed on the skin in order to increase blood flow (Ghorebaghian, Karimi, Vafaian, & Tabrizi Naimi, 2010).

#### 1.2.4 Leech Therapy

It refers to a medical bleeding method which has a special place in traditional medicine due to the style of bleeding and Hirudin which inserts from the leech to the patient’s body (Farshad, 2013).

#### 1.2.5 Fasd

It refers to another bleeding method in traditional medicine. It leads to the repulsion of a lot of poisons and other superfluous materials which increase in the blood.

### 1.3 Aims of the Study

#### 1.3.1 Major Purpose

The main purpose of this study is to determine knowledge and attitude of students of nursing towards Iranian traditional medicine in medical universities in Tehran.

#### 1.3.2 Minor Purposes

-Finding demographic features of the students (age, gender, ethnicity, number of semesters they had passed)
-Determining the participants’ general knowledge about Iranian traditional medicine.
-Determining the participants’ general attitude towards Iranian traditional medicine.
-Determining the participants’ attitude towards incorporating courses on traditional medicine in their curriculum.
-Determining the relationship between the participants’ general knowledge about Iranian traditional medicine and their demographic features.
-Determining the frequency of different barriers to using Iranian traditional medicine in Iran’s heath system from students’ viewpoints.
-Determining the frequency of students’ participation in Iranian traditional medicine training courses.

## 2. Method

This descriptive, cross-sectional study examined the knowledge and attitudes of first to third year of students of nursing in 2012-2013. Considering the knowledge and attitude of participants in other similar studies (70%) and considering the accuracy (d = 0.05) and reliability (95%) and z = 1.96, in the present study, 300 participants were used as the sample. A questionnaire including five sections (demographic information, general knowledge about Iranian traditional medicine, general attitude towards Iranian traditional medicine, availability of resources and barriers to Iranian traditional medicine) was used for data collection. This questionnaire has been designed by traditional medicine researchers through modeling similar questionnaires employed by Halterman, Sierpina, Sadoski and Sanders, (2009), Naghibi et al. (2009), and “medicine integrative attitude questionnaire”. It should be mentioned that for assessing students’ knowledge of traditional medicine, due to the extent of this science, we made use of the recommendations of WHO regarding traditional medicine (WHO, 2002). The face validity of the questionnaire was confirmed by five expert researchers in traditional medicine. Its internal reliability was calculated using Cronbach’s alpha and factor analysis. It was piloted by using 20 participants who were similar to the research participants in terms of demographic features. This pilot study helped the researchers remove probable problems of the questionnaire. The five sections of the questionnaire are as follows: 1) demographic features of the students (age, gender, number of semesters they had passed and their ethnicity; 2) general knowledge about Iranian traditional medicine including 14 yes/no questions which determined the status of Iranian traditional medicine in Iran and across the world as well as the knowledge of treatment methods and resources of traditional medicine; 3) general attitude towards Iranian traditional medicine including 26 questions whose answers were ranked on a Likert scale from 1 (strongly disagree) to 5 (strongly agree). Some of the questions are as follows: investigating attitudes towards the status and efficacy of traditional medicine, the effects of taste and life style such as diet on health, holistic viewpoint, encouraging treatment force, the placebo effect of this method and suggestions for including traditional and complementary medicine in university curriculums; 4) the barriers to using traditional medicine in Iran was again ranked on a Likert scale from 1 to 5. These barriers included lack of evidence, unavailability of experts with valid educational degrees, lack of insurance coverage, being time-consuming, lack of staff training, lack of appropriate equipments, and legal issues; 5) resources of traditional medicine were ranked from 1 (not useful at all) to 2 (not useful), 3 (I have no idea), 4 (are useful) to 5 (are very useful). These resources included the Internet, related books, lectures, practical experience in clinical environment, observing treatments through traditional medicine, and the research papers reporting the results of clinical experiments. In addition, at the end of the questionnaire, the respondents’ participation in at least one training course on traditional medicine and at least one training course on complementary medicine was questioned which required just a yes/no answer. Before distributing the questionnaires, the researchers introduced themselves and explained the plan and the questions to the students. The students, who accepted to participate, signed the consent forms for medical ethics of Tehran University of Medical Sciences and filled in the questionnaires. The questionnaires were anonymous and contained the related identity codes. Participation in the plan was voluntary without manipulating any power.The results obtained from the questionnaire were analyzed using the SPSS software. For data analysis, different descriptive statistics (mean, absolute frequency, relative frequency) and inferential statistics (chi-square test and correlation) were employed. 0.05 was considered as the level of significance.

## 3. Results

From among 300 participants, 4 cases did not answer the questionnaire completely, and they were omitted. 71.6 percent of the participants were female and 25.7 percent of them were male. Their age range was 21.17 ± 3.65 (Table 1).

**Table 1:** Age

N Valid	286
Missing	10
Mean	21.17
Median	21.00
Std Deviation	3.652

Regarding their ethnicity, 54.1 percent of them were Fars and 15.5 percent of them were Turks. 33.8 percent of them had knowledge about the status of traditional medicine in Iran (Table 2 and Table A1 in Appendix A) and 19.6 percent of them had knowledge about the status of traditional medicine in the world (Table 2 and Table A2 in Appendix A). Their familiarity with the sources of traditional medicine was 25.9% (Table 2 and Table A3 in Appendix A), classification of tastes and moods was 26% and the effects of factors such as weather, amount of being sleep or awake, and rest and movement was 37.2%. Their knowledge about reasons of diseases and their symptoms from traditional medicine standpoint and the ways of remaining healthy was 21.6%. their level of familiarity with phytotherapy was 48.3%, with diet was 36.7%, with complementary medicine was 23.8%, with cupping was 72.3%, with leech therapy was 62.2%, with massaging was 60.5% and with fasd was 23.0% (Table 2).

**Table 2:** The results of the questionnaire

Knowledge About	YES	NO
The status of traditional medicine in Iran	33/8%	66/2%
The status of traditional medicine in the world	19/6%	4%/80
Resources of traditional medicine	25/9%	74.1%
classification of tastes and moods in traditional medicine	26%	74%
effects of factors such as weather, amount of being sleep or awake	37/2%	62/8%
Reasons of diseases and their symptoms	37/2%	62/8%
The ways of maintaining health from the standpoint of traditional medicine	21/6%	78/4%
Phytotherapy from the standpoint of traditional medicine	48/3%	51/7%
Diet	36.7%	63/3%
Complementary medicine	23/8%	76/2%
Cupping	72/3%	27/7%
Leech therapy	62/2%	37/8%
Message	60/5%	39/5%
Fasd	23%	77%

Total	36/8%	63/2%

The general knowledge of the participants about the status of traditional medicine in Iran was 36.8% (below 50% is considered as low knowledge) (Table 2).

There was no statistically significant difference between sex and general knowledge of the participants about traditional medicine (p = 0.11), (Table 3) while there was a statistically significant difference between their age and knowledge of traditional medicine (p = 0.00) (Table 4). There was also a significant difference between the number of semesters they had passed and their general knowledge of traditional medicine (p = 0.00) (Table 5).

**Table 3:** Knowledge* sex

	Value	df	Asymp. Sig. (2-sided)
Pearson Chi-Square	37.033a	28	.118
Likelihood Ratio	38.825	28	.084
N of Valid Cases	293		

Note: df=degree of freedom.

**Table 4:** Knowledge* age

	Value	df	Asymp. Sig. (2-sided)
Pearson Chi-Square	3.358E2^a^	154	.000
Likelihood Ratio	246.732	154	.000
Linear-by-Linear Association	.003	1	.954
N of Valid Cases	283		

Note: df=degree of freedom.

**Table 5:** Knowledge* the number of semesters they had passed

	Value	df	Asymp. Sig. (2-sided)
Pearson Chi-Square	1.385E2^a^	56	.000
Likelihood Ratio	81.699	56	.014
N of Valid Cases	293		

Note: df=degree of freedom.

There were no statistically significant differences between participants’ attitudes towards traditional medicine and their age and also between participants’ attitudes towards traditional medicine and the number of semesters they had passed.

Based on the results, cupping, leechthrapy and message are the factors which were optimal from students perspective. However, the other two dimensions namely fasd and complementary medicine from students perspectives has been assessed weakly.

The attitude of students towards Iranian traditional medicine with factors such as (weather and sleep and rest can be important to health, lifestyle modifications including diet therapy is effective, the effectiveness of traditional medical therapies is due to holistic view for humans, effects of supplementation are mainly due to the placebo effect, the impact should only be judged based on scientific research and evidence-based methods, limitations of conventional methods are the main drawbacks of traditional medicine, only Less informed people use traditional medicine, clinical care should be a combination of traditional and conventional medicine, some methods of traditional medicine must be officially licensed, Iranian traditional medical courses and complementary medical courses should be included as part of my academic curriculum, knowledge about traditional medicine is important for my future) were assessed (Table 6).

**Table 6:** Attitude of nursing students at universities of Tehran towards Iranian traditional medicine

Attitude	Totally agree	agree	No idea	disagree	Totally disagree
Traditional Iranian medicine has its proper status	5.4%	15.5%	37.8%	29.7%	11.5%
Factors such as weather and sleep and rest can be important to health	32.4%	52.7%	10.1%	3.4%	0.7%
Lifestyle modifications including diet therapy is effective	38.4%	45.9%	11.5%	1.4%	0.7%
The effectiveness of traditional medical therapies is due to holistic view for humans	3.4%	18.9%	70.9%	4.1%	1.4%
Effects of supplementation are mainly due to the placebo effect	2.7%	6.8%	77.7%	6.8%	2.0%
The impact should only be judged based on scientific research and evidence-based methods	11.5%	37.8%	37.8%	9.5%	1.4%
limitations of conventional methods are the main drawbacks of traditional medicine	5.4%	29.7%	43.7%	18.2%	2.7%
Only Less informed people use traditional medicine	2.7%	7.4%	29.7%	39.7%	19.6%
Clinical care should be a combination of traditional and conventional medicine	25.7%	39.7%	27%	4.1%	2%
Some methods of traditional medicine must be officially licensed	34.5%	37.2%	20.9%	4.1%	2%
Iranian traditional medical courses should be included as part of my academic curriculum	22.3%	45.3%	19.6%	7.4%	2%
complementary medical courses should be included as part of my academic curriculum	26.4%	32.4%	27.7%	8.1%	2%
Knowledge about traditional medicine is important for my future	25%	39.2%	23.6%	1.4%	1.4%

Their attitude towards including Iranian traditional medicine in their curriculum was 3.81 (based on the Likert scale from 1 to 5); therefore, it is positive (quite positive 4–5, positive 3–4, I have no idea 2–3, and negative 1–2). Their attitude towards including complementary medicine was positive (3.75) (Table 7). Their general attitude towards Iranian traditional medicine was positive too (3.5).

**Table 7:** Attitudes towards complementary medicine and traditional medicine

	Attitudes towards complementary medicine	Attitudes towards traditional medicine
Valid	287	287
Missing	9	9
Mean	3.75	3.81
Median	4.00	4.00
Std. Deviation	1.013	.947

96.6% of the participants had not passed any formal training course on Iranian traditional medicine (Table A4 in the appendix A) and 97.9% of them had not any course on complementary medicine (Table A5 in the appendix A).

Regarding the questions about barriers, the biggest barrier in participants’ viewpoint was unavailability of the experts with valid academic degrees (3.77) and the smallest barrier was that it requires a lot of time (3.22). Other barriers included lack of evidence for using this medicine, lack of insurance coverage and lack of training to staff which were 3.46, 3.72, and 3.77 respectively.

Participants believed that the Internet was quite informative for them about traditional medicine (4.14 based on the Likert scale from 1-5), lectures were also useful (3.88), textbooks were also quite useful (4.05), the results of research papers on clinical experiments were quite useful (4.3) and the practical experience of traditional medicine was again quite useful (4.39) (Figure 3).

**Figure 1:**
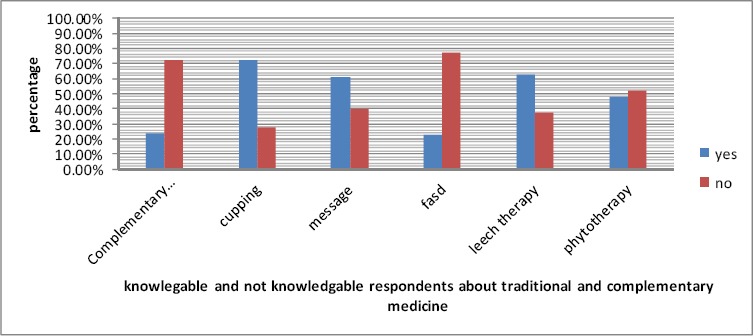
Percentage of knowledgeable and not knowledgeable respondents about traditional and complementary medicine

**Figure 2:**
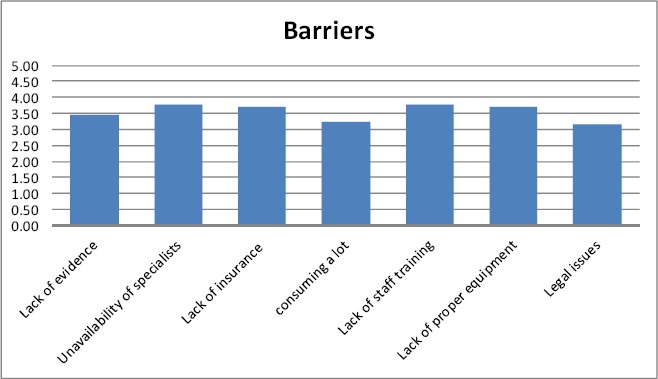
The frequency of each barrier in using Iranian traditional medicine from student’s perspective

**Figure 3:**
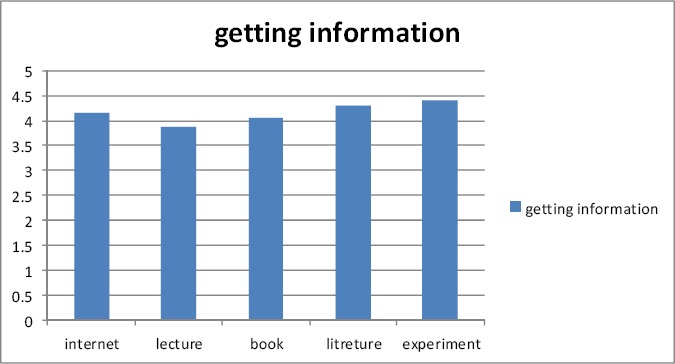
The resources of getting information about traditional medicine

## 4. Discussion and Conclusion

The results of this study, which for the first time assessed the knowledge and attitude of the nursing students in Iran towards traditional and complementary medicines, revealed that although the nursing students had insufficient knowledge about traditional medicine, they had a positive attitude towards its learning and application. There was no statistically significant difference between gender and general knowledge of the students about traditional medicine (p = 0.11) while there were significant differences between general knowledge of the students about traditional medicine and their age and also the number of the semesters they had passed (p = 0.00). There was no significant difference between their attitudes towards traditional medicine and the number of the semesters they had passed and also their age.

Considering the participants’ lack of presence in training courses on traditional medicine, it seems that including courses on traditional medicine and complementary medicine in the students’ curriculum helps them improve the treatment process of their patients. The results of the study by Zeighami (2006) on the nurses working in hospitals in Kerman also revealed that the level of knowledge of the nurses about complementary medicine was low; however, the majority of them were very interested in enhancing their knowledge about it. In addition, there was a weak, positive correlation between the nurses’ knowledge, attitudes and their application of complementary medicine. In another study, Mashhadi (2010) investigated the level of knowledge and attitudes of the students of medicine towards traditional medicine in universities of Tehran. The results revealed that the participants’ knowledge was rather high (60%) and their attitude was quite positive (80%). Despite the average knowledge of medicine students about complementary medicine, their attitude towards its learning was high (Mashhadi, 2010). Naghibi et al. (2009) also examined the attitudes of medicine students and assistants towards Iranian traditional medicine. The results of their study revealed that most of the participants had no knowledge of the principles of traditional medicine and its treatment methods, they did not apply it, and they did not advise it to their patients. However, half of the participants were willing to learn it and believed in it. In this study, medicine students had more knowledge of the treatment methods of Iranian traditional medicine compared with the assistants (Naghibi et al., 2009). Another study in Canada compared the viewpoints of the students of medicine with the viewpoints of the students of pharmacy, nursing and physiotherapy about complementary medicine. The results revealed those participants who had experienced the methods of complementary medicine before, believed that it is more useful. However, knowledge of the medicine students was lower compared with knowledge of the students of other fields. The students of medicine and pharmacy insisted on scientific confirmation of complementary medicine before its application more than the students of other fields (Baugniet et al., 2000). The results of the study by Xue et al. (2008) on the students of Hong Kong nursing university also confirmed personal application and advice of complementary medicine to others. 80% of the participants of this study had employed the methods of complementary medicine at least one time. Also, the attitude of the participants regarding the use of complementary medicine in initial clinical services revealed their interest. Most of the participants of this study referred their patients to specialists in complementary medicine. Their reason for this was either the patients’ own tendency or their lack of satisfaction with the results of usual medicine (Van Haselen, Reiber, Nickel, Jakob, & Fisher, 2004). In research by Tiralongo and Wallis (2008) the majority of the pharmacy students (95%) believed that pharmacists should be able to give enough information to patients about complementary medicine. They believed they are really in need of information about complementary medicine. The results of all these studies are in line with findings of the present study. Nurses’ more knowledge about traditional medicine revives ancient medical heritage and also helps make other people familiar with this medicine.

## Appendix

**Table A1:** Frequency of the responses to the question of “I have knowledge about the status of traditional medicine in Iran”

	frequency	percent	Valid percentage
no	196	66.2	66.2
yes	100	33.8	33.8
total	296	100	100

**Table A2:** Frequency of the responses to the question of “I have knowledge about the status of traditional medicine in the world”

	frequency	percent	Valid percentage
no	2.38	80.4	80.4
yes	58	19.6	19.6
total	296	100	100

**Table A3:** Frequency of the responses to the question of “I have knowledge about the resources of traditional medicine”

	frequency	percent	Valid percentage
no	218	73.6	74.1
yes	76	25.7	25.7
blank	2	0.7	100
total	296	100	

**Table A4:** Frequency of the responses to the question of “I have passed at least one course on traditional medicine”

	frequency	percent	Valid percentage
no	280	96.6	96.6
yes	10	3.4	3.4
blank	6	2	
total	296	100	100

**Table A5:** Frequency of the responses to the question of “I have passed at least one course on complementary medicine”

	frequency	percent	Valid percentage
no	284	97.9	97.9
yes	6	2.1	2.1
blank	6	2	
total	296	100	100
